# Supplementary material for: Are carbon emissions trading and green financial instruments synergistic? -Comprehensive quantitative research based on content analysis
Source: PLoS One. 2024 Mar 7;19(3):e0298601. doi: 10.1371/journal.pone.0298601 (PMC10919601; doi:10.1371/journal.pone.0298601)
Supplement: S1 Data — (ZIP) [file pone.0298601.s001.zip › Data/地级市Z_F工作报告.zip!/┴╔─■/┴╔╤⌠╩╨/└┤╫╘╣1⁄2╓┌║┼í╛┬φ┐╦╩2╛▌═°í┐_2019.docx]

各位代表：

　　 我代表市政府向大会报告工作，请予审议，并请市政协委员和列席会议的同志提出意见。

　　一、2018年工作回顾

　　刚刚过去的2018年，是外部环境复杂严峻、改革发展稳定任务繁重的一年，更是辽阳发展史上具有里程碑意义的一年。一年来，在省委、省政府和市委的正确领导下，市政府团结带领全市人民，全面贯彻习近平新时代中国特色社会主义思想和党的十九大及十九届二中、三中全会精神，深入落实习近平总书记在辽宁、辽阳考察时和在深入推进东北振兴座谈会议上的重要讲话精神，坚持稳中求进工作总基调，牢固树立新发展理念，按照市委十二届六次、七次、八次全会和市十六届人大一次会议部署要求，以供给侧结构性改革为主线，着力打好三大攻坚战，加快改革开放步伐，推动“四个辽阳”建设迈上新台阶，全市呈现经济总体向好、民生持续改善、社会和谐稳定的良好局面。

　　预计，地区生产总值实现850亿元，增长5.2%；一般公共预算收入实现89.4亿元，增长10.8%；固定资产投资增长1.5%；规模以上工业增加值增长9%；社会消费品零售总额实现304亿元，增长5.6%；城乡常住居民人均可支配收入分别实现32310元和14895元，均增长7%。

　　一年来，我们重点抓了以下七项工作。

　　（一）抓工业强项目，转型升级步伐加快

　　持续调整产业结构。着力推进“两大千亿元产业基地和一大产业集群”建设，营业收入分别增长35%、8%和20%。芳烃基地入选“2018中国化工园区30强”、获批“辽宁省知名品牌创建示范区”，辽化大技改项目全面投产运行，原油加工能力大幅提升。忠旺铝合金精深加工等6个项目全面开（复）工，新型高强建筑模板、全铝轿车车身、全铝智能家具等重点项目投入运行。天一航空密封件等16个项目开（复）工，富奥弹簧、施耐德等企业产值快速增长，装备制造及汽车零部件产业集群保持良好发展态势。加快钢铁、水泥产业转型发展，6户“地条钢”企业成功转型，淘汰35万吨水泥落后产能，辽阳联合钢铁有限公司满负荷运行。3户企业的项目和产品分别中标国家工业强基工程、荣获中国工业设计优秀产品奖和中国国际新材料产业博览会金奖，3户企业获批省“‘两化’融合管理体系贯标试点”，新增6户省级服务型示范企业和工业设计中心。

　　积极推动科技创新。深化政产学研合作，促成科技成果转化38项。创新科技金融，发放贷款近6000万元。忠旺国家重点实验室建设稳步推进，华信钢铁共性技术创新中心中试基地开工建设。奥克集团、三三工业晋升国家技术创新示范企业，培育省级产业技术创新平台和产业技术创新联盟8家。新增高新技术企业39家、科技型中小企业108家。着力推进创新创业，高新区与沈阳工业大学共建的国家级孵化器投入运行，培育了4家国家级知识产权优势企业和2家星创天地，选派科技特派员31名。

　　倾力帮扶实体经济。出台一系列支持民营经济发展优惠政策，全面落实各项减税降费措施，为企业减负86亿元。扩大直供电准入范围，为企业节省用电成本9100万元。集中开展盘活存量建设用地专项整治行动，盘活闲置土地25.8公顷。运用股权质押等方式，帮助企业融资183亿元。深入实施企业培育工程，完成“个转企”516户、“小升规”27户。

　　深入开展“项目年”活动。全力推进121个亿元以上重点项目建设，竣工投产42项，完成97个“四个辽阳”建设重点项目年度任务。争取上级资金90亿元，融资担保能力达到100亿元。千方百计引资引智，开展“走出去、引进来”招商活动200余次，促成46个新签约项目落地，国内到位资金132.4亿元，实际利用外资5460万美元，培养、引进高层次专家团队9个。

　　（二）抓三产重特色，质量档次稳步提升

　　推动生产性服务业专业化集聚化。持续壮大金融业，金融机构本外币各项贷款余额1597亿元，增长16.8%，比年初增加230亿元，金融业税收占全市10.7%，同比提高2.7个百分点。创新发展电子商务，先后与天猫、京东等大型电商平台签订合作协议，3个电子商务产业园全部投入运行，网络交易额实现30亿元，增长25%。加快发展现代物流业，肉菜流通追溯体系建设有序推进，新增农产品直销网点30个，出口俄罗斯跨境冷链班列持续运行。

　　推动生活性服务业优质化便利化。弓长岭全时全域旅游目的地正在加速形成，佳兆业铂域酒店等一批项目投入使用。推动佟二堡千亿元市场建设，举办首届皮装裘皮服装国际博览会，年交易额200亿元左右。实施国家旅游“厕所革命”新三年行动计划。深入推进“后备箱”工程，培育12家旅游购物店和28种特色商品。旅游总收入实现225亿元，增长12.7%。大力发展医养结合养老产业。共享单车等分享经济蓬勃兴起。首批认定“真乐”食品、“襄元堂”医药等17家“辽阳老字号”，被省认定3家“辽宁老字号”。引进何氏眼科、地利生鲜等知名品牌181个。持续繁荣假日经济、夜经济，成功举办冰雪节、啤酒节等系列活动，拉动多样式消费需求。

　　（三）抓农业惠农村，乡村振兴取得实效

　　深入推进国家现代农业示范区建设。10个现代农业示范区（带）建设成效显著，实施123个乡镇现代农业精品项目，太子河东宁卫乡荣获国家级农业产业强镇示范建设单位，三禾农业观光园进入全国休闲农业和乡村旅游精品景点线路。

　　加快推进农业供给侧结构性改革。在全省率先开展农村土地承包经营权确权登记颁证，完成集体产权制度改革清产核资任务。加快土地流转，新发展千亩以上土地股份合作社10家。调减玉米种植面积3万亩，粮食产量101万吨。建成农家科普大院35个、科技小院10个。实施10个“全程机械化+农事服务中心”升级工程，耕种收综合机械化率85%。实施23个农业产业化重点项目，培育了13家农事龙头企业。“灯塔大米”“灯塔葡萄”荣获国家农产品地理标志。秸秆综合利用率达到85%。非洲猪瘟疫病和松材线虫病防控取得阶段性成果。

　　加快完善农业农村基础设施。建设高标准农田7.5万亩。完成灯塔和浑沙灌区年度建设任务，改善灌溉面积4.5万亩。44项水库移民工程全面竣工。解决了1.1万人安全饮水问题。完成39个村“气代煤”改造。大中修、改扩建各级公路468.4公里，“四好农村路”建设全省领先。样子岭隧道投入运行。108个“升级版”美丽乡村示范村全部建成。农村人居环境整治取得一定成效。

　　大力发展县域经济。全面落实县域经济发展三年行动计划。完成县乡财政管理体制改革，36个乡镇全部建立金库。扶持114个集体经济薄弱村建设。培育壮大“块状经济”，实施特色小镇基础设施和主导产业项目39个，小北河袜艺小镇列入省首批产业特色小镇创建名单，产业整体规模跃居全国第4位。

　　（四）抓改革促开放，发展活力显著增强

　　全面优化营商环境。在全省率先颁布实施营商环境建设地方性法规，推进“办事难”和“七病一症”专项整治，社会信用体系建设在全国动态监测的262个地级市中位列20位左右。取消、下放行政审批权限45项，减少审批环节351个，企业开办时间缩减至2.5个工作日以内，审批时限全省最短。深入推进“双随机、一公开”，常态化跨部门联合检查机制基本形成。“一网、一门、一次”政务服务改革不断深化，公共资源交易平台全面建成，政务服务网和服务平台上线运行，审批集中办理率达到95%，不动产登记实现集中办理，118项审批事项实现“最多跑一次”，34项实现“不见面”审批。

　　深化重点领域改革。完成全省厂办大集体改革试点年度任务，稳妥处置4户“僵尸企业”。国税地税征管体制改革基本完成。多措并举化解政府债务56亿元，风险总体可控。加快推进“证照分离”“多证合一”等商事制度改革，新增市场主体2.4万户。深化公立医院综合改革，实施分级诊疗制度，破除以药补医机制，实现基本药物制度全覆盖。全面实施“河长制”，市县乡村四级责任体系得到有效落实。完成市级政府机构和公益性、经营性事业单位改革任务。产业园区、供销、农垦等领域改革有序推进。

　　提升对外开放水平。4家企业建立海外仓，培育4个国家和省级外贸转型升级基地，新增2家省级外贸综合服务和出口名牌企业。参加首届中国国际进口博览会，签约额超5亿元。积极响应“一带一路”倡议，推动19家企业开展境外投资合作。全面落实沈阳经济区辽阳建设发展“三年攻坚计划”，入股“沈阳经济区产业基金”，积极参与“沈阳港”建设。与江苏南通签订了对口合作框架协议，已有8个项目开展合作。

　　（五）抓城建优环境，城乡面貌持续改善

　　加强城市基础设施建设。宏伟路全线贯通，解放路、北工街、新华路北段等5条城市干道竣工通车，河东交通综合枢纽二期开工建设，新城配套设施和服务功能日益完善，新老城区融合更加紧密。徐往子游园建成并对外开放。实施一批绿化美化亮化工程，燃气、供暖、电力等基础设施不断完善。

　　提高城市管理水平。深入推进城管执法体制改革，实现执法重心下移。发挥数字化城市管理平台作用，实行网格化管理考核。实施城市市容和环境卫生管理条例，集中开展集贸市场、环境卫生、露天烧烤等系列治理行动，市容市貌持续改善。大力整治交通秩序，渠化改造6个道路交叉口，机动车礼让斑马线蔚然成风。全国文明城市创建成果得到巩固。

　　不断优化生态环境。全面启动国家森林城市创建工作，人工造林、封山育林6.2万亩，绿化村屯128个。严控秸秆焚烧，淘汰燃煤锅炉162台，空气质量达标率81.4%。扎实开展太子河生态治理工程，加强饮用水源地环境保护，拆除汤河饮用水源地一、二级保护区内违规建筑，开展新开河等重点河流治理，城镇污水处理厂全部达到一级A排放标准。推进土壤环境质量监测网络建设，启动重点行业企业用地污染状况基础信息采集工作。治理农村面源污染，畜禽养殖场粪污处理设施化率达到89%。基本完成“大棚房”问题清理整治任务。双河及周边地区生态修复工程全面启动，环湖路开工建设。领导干部自然资源资产任中审计工作初见成效。完成中央环保督察和“回头看”整改序时任务。

　　（六）抓民生增福祉，社会大局保持稳定

　　如期完成20项重点民生实事，群众满意度进一步提升。

　　强力推进脱贫攻坚。实施“五个一批”工程，稳定脱贫1986人，摘帽12个贫困村。精准医疗扶贫工作受到国务院通报表扬。改造农村危房1063户，工作经验全省推广。

　　着力提升社会保障能力。新增城镇就业1.7万人，城镇登记失业率始终控制在3.5%以内。城镇居民医保财政补助标准提高至490元，新农合人均筹资标准提高至700元，城乡低保标准分别提高7.8%和7.5%，农村特困人员供养标准提高32.7%。多渠道筹措资金，确保了养老金按时足额发放。开展已售商品房产权证办理问题专项行动，为11688户居民解决了办证难题。实施棚改项目12个，改造老旧小区20个。分配公租房519套，发放廉租房租赁补贴3812户。

　　大力发展各项社会事业。协作化办学成效显著，课后服务改革、“三通两平台”建设走在全省前列。市一高中、弓长岭高中分别被评为辽宁省特色高中和示范高中。10家乡镇卫生院房屋标准化建设达到省级要求，市九院与弓长岭区中心医院完成整合，在全国率先实行“120”院前急救免费服务，结核病等重大传染性疾病得到有效防控。成功获批全国居家和社区养老服务改革试点市。开展第七届全民读书节、“河东之夏”、《花木兰》芭蕾舞剧、全国学雷锋先进典型走进弓长岭等各类宣传文化活动，市场化运作一系列体育赛事。完成了历史文化街区修缮、明城墙和魁星楼遗址保护工程，创建历史文化名城通过国家评估。第四次全国经济普查扎实推进。国防动员、人防和民兵预备役建设进一步加强。气象、防震减灾、档案、老龄、慈善、红十字、妇女儿童、残疾人和地方志等事业取得新进步。

　　强化社会综合治理。全方位构建社会治安防控体系，圆满完成重大警卫安保任务。深入开展扫黑除恶专项斗争，成功打掉涉黑涉恶团伙43个。全面落实信访工作责任制，超额完成减存控增年度目标。安全生产总体形势保持平稳，事故起数和死亡人数分别下降4.1%和2.2%。群众饮食用药安全得到有效保障，产品质量安全水平进一步提升。全面规范清理各类社会组织。民族宗教、国家安全、保密、外事侨务、涉台事务、应急管理等工作取得新成效，社会大局保持和谐稳定。

　　（七）抓自身转作风，政府效能全面提升

　　主动接受市人大及其常委会法律监督、工作监督，向市人大常委会报告工作10次，办理人大代表议案和建议155件。自觉接受市政协民主监督，办理政协建议案和提案264件。

　　全面推进依法行政。深入实施“七五”普法，法律服务和法律援助不断加强。扎实开展立法工作，提请市人大常委会审议2件地方性法规草案。加强审计监督，财经秩序得到有效维护。完成各级政府网站集约化升级改造，信息公开水平进一步提升。

　　切实转变工作作风。推进“两学一做”学习教育常态化制度化，全面开展“解放思想推动高质量发展大讨论”，持续深化“五包五促”活动。实施“重强抓”专项行动，促进了业务作风“双提升”。12345市长热线与8890政务便民服务平台实现双线并行，群众和企业诉求渠道更加畅通。切实加强政府系统党风廉政建设，清正清廉风气正在形成。

　　各位代表！过去一年取得的成绩来之不易，是省委、省政府和市委正确领导、科学决策的结果；是全市广大干部群众团结奋斗、开拓进取的结果；是市人大和市政协有效监督、大力支持的结果；是驻军部队，中央、省驻我市单位，社会各界人士、外来投资者和建设者，各民主党派、人民团体辛勤工作、关心支持的结果。在此，我代表市政府，向参与辽阳经济社会建设的全市人民和各界人士表示衷心的感谢！

　　各位代表！在总结成绩的同时，我们也清醒地看到，全市经济社会发展还存在诸多不平衡不充分问题。一是产业结构不尽合理，经济发展质量效益还不高，重大项目特别是新建项目、产业项目少，产业链条不完善，招商氛围不浓厚，新兴产业有效支撑不足。二是创新能力不强，高新技术企业偏少，高层次技术和管理人才匮乏，企业研发投入不足，核心竞争力有待进一步提升。三是民生领域仍有不少短板，城市管理、社会治理、生态环保等方面与广大人民群众的期盼还有差距，政府性债务、养老金缺口等风险压力较大，教育资源不足、水平不高，养老事业产业发展滞后。四是干部队伍思想观念、精神状态和工作作风与新时代的要求还有差距，政府服务能力还有待提高。这些问题，我们将在今后工作中认真加以解决。

　　二、2019年重点工作安排

　　2019年，是新中国成立70周年，是全面建成小康社会的关键之年，是全面贯彻落实习近平总书记在辽宁、辽阳考察时和在深入推进东北振兴座谈会议上重要讲话精神的开局之年，是全面推进“四个辽阳”建设的重要之年。当前，经济面临下行压力，危和机同生并存。从国际形势看，贸易保护主义抬头，中美贸易摩擦产生的影响正在逐步显现，世界经济增长不确定性明显上升。从国内形势看，虽然宏观经济运行总体保持平稳，但投资、消费等内需动力增长有所放缓，一些企业出现暂时性发展和经营困难。从省内形势看，受传统产业发展模式影响和体制机制制约，经济发展内生动力不足，结构调整任务较重。

　　压力就是动力，挑战蕴含机遇。我们看到，党中央、国务院审时度势，化被动为主动，持续优化产业结构，坚定不移推动高质量发展，经济运行保持在合理区间。我们看到，习近平总书记在辽宁、辽阳考察时和在深入推进东北振兴座谈会议上，明确提出了“六项要求”，党中央、国务院将进一步出台支持东北全面振兴的政策举措，省委、省政府也制定了促进新时代辽宁全面振兴的“1+8”意见和支持民营经济发展的3个文件，政策含金量之高、支持力度之大前所未有，这必将为辽阳经济发展注入新的动力和活力。我们看到，“四个辽阳”建设引领全市经济社会持续向好，发展潜能不断集聚，特别是习近平总书记到辽阳考察，极大地鼓舞了全市上下干事创业激情，辽阳必将进入史无前例的发展新阶段。只要我们坚定信心、抢抓机遇、开拓创新、乘势而上，就一定能够战胜前进道路上的艰难险阻，开创新时代辽阳全面振兴发展新局面。

　　今年政府工作的指导思想是：高举习近平新时代中国特色社会主义思想伟大旗帜，深入贯彻党的十九大和十九届二中、三中全会精神，全面落实习近平总书记在辽宁、辽阳考察时和在深入推进东北振兴座谈会议上的重要讲话精神，统筹推进“五位一体”总体布局，协调推进“四个全面”战略布局，坚持稳中求进工作总基调，按照中央、省委经济工作会议和市委十二届九次全会部署，践行新发展理念，落实高质量发展要求，以供给侧结构性改革为主线，深化市场化改革、扩大高水平开放，加快建设现代化经济体系，大力实施乡村振兴战略，进一步做好“六稳”工作，实施新时代辽阳全面振兴“1+10”意见，多措并举招才引智，着力打造产业生态圈，高标准融入沈阳经济区，全力保障和改善民生，为全面建成小康社会收官打下决定性基础，以新气象新担当新作为开创“四个辽阳”建设新局面，谱写新时代全面振兴发展新篇章。

　　今年经济社会发展主要预期目标是：地区生产总值增长6.5%左右，一般公共预算收入增长6.5%左右，固定资产投资增长8%左右，规模以上工业增加值增长8%左右，社会消费品零售总额增长6%左右，城乡常住居民人均可支配收入分别增长6.5%左右和7%左右，城镇登记失业率控制在4.4%以内。

　　我们要突出抓好以下八个方面工作：

　　（一）坚持“项目为王”，夯实新时代经济发展核心支撑

　　牢固树立以经济建设为中心的思想，把项目建设作为经济工作的核心，以项目论英雄，建立健全全员抓招商、全员抓项目的考评体系，持续增强经济发展后劲。

　　深化“项目年”活动。全面推行重点项目“服务秘书”和专题会议制度，实施137个投资亿元以上项目建设。围绕重点产业、重点区域，积极谋划、包装项目，增强项目储备。

　　全面做好要素保障。积极主动做好项目、政策、资金“三争取”工作，到位资金95亿元以上。规范开展政府和社会资本合作。有效争取省地债资金。加强银企对接，新增贷款200亿元。深入开展盘活存量建设用地专项整治行动，为项目建设腾出空间。

　　加快产业园区创新发展。完善高新区、经济开发区体制机制，实行“管委会+公司”模式，激活发展内生动力。加快水、电、气等配套基础设施建设，全力破解制约项目建设的瓶颈问题，切实增强承载能力。落实“飞地经济”政策，促进区域间项目布局优势互补。

　　大力开展“双招双引”。完善并实施新一轮招商引资政策。进一步明确招商方向，密切跟踪产业发展动态，提高招商质量和成功率。发扬“四千”“五皮”精神，深入开展全员招商、精准招商、专业招商、产业链招商、以商招商，引进更多战略投资者。坚守契约精神，全面兑现招商承诺。国内到位资金和实际利用外资均增长10%。落实集聚人才优惠政策，加大招才引智力度。

　　（二）深化改革开放，打造新时代营商环境“金字招牌”

　　以优化营商环境为基础，全面深化改革，提升开放合作水平。

　　持续优化营商环境。全力做好“简”“减”两篇文章，审批流程再简化，审批环节再减少，审批时限再压缩15%，审批事项再下放20%。深入推进“双随机、一公开”，放开前端、强化中端、管住后端。创新“互联网+政务服务”，推动“一网、一门、一次”改革向纵深发展，进一步提高审批服务事项集中办理率和网上可办率，实现市县乡网上审批全覆盖。整合支持企业发展的减税降费、研发投入、首台首套等各项政策，为企业快速发展营造更好的政策环境。开展营商环境第三方评估和结果运用，强力推进专项整治行动，加大宣传、暗访和曝光力度，建立健全联席会议、多方参与、追责问效的长效机制。加强政府诚信建设，争创全国第二批社会信用体系建设示范城市。坚持定期联系企业家制度，依法保护民营企业权益，保障企业知情权、话语权和参与权。

　　毫不动摇地巩固和发展公有制经济。支持辽化公司进一步扩能改造，延伸精细化工产业链。培育壮大庆化军民融合产业园，鼓励“民参军”“军转民”。深化国资国企改革，完成厂办大集体改革试点、僵尸企业处置和“三供一业”分离移交任务。积极推进混合所有制改革，推动国资国企监管由管资产向管资本转变。

　　毫不动摇地鼓励、支持、引导非公有制经济发展。加快民营经济发展改革示范市建设。设立1亿元民营企业创新发展基金和2亿元“助保贷”风险补偿金，帮助企业解决融资难题。设立新兴产业创业投资引导基金，做实做强市级融资担保平台，发挥普惠金融服务中心作用，加大金融支持中小微企业力度，推动金融资本更多投向实体经济。充分利用资本市场，加快推进4户企业上市进程。深入实施企业培育工程，“个转企”400户，“小升规”18户，“规升巨”5户，引导忠旺集团、奥克集团、科隆精化、三三工业、瑞兴化工等民营企业进一步做大做强，加快形成大企业“顶天立地”、中小企业“铺天盖地”的发展态势。支持行业协会和商会健康发展。弘扬企业家精神，秉承辽商优良传统，建设新时代高素质专业化企业家队伍。

　　推进重点领域改革。全面完成政府机构改革。防范和化解区域性金融风险，严格政府债务限额和预算管理，建立健全风险预警机制，化解政府债务48亿元。深化基本公共服务领域财政事权和支出责任划分改革，全面落实个人所得税改革政策。推进公立医院薪酬制度和医院按病种收付费改革，深入实施分级诊疗制度。加快商事、供销等领域改革步伐。

　　构建开放合作高地。积极响应“一带一路”倡议，鼓励引导骨干企业开拓国际市场。加强与军贸企业合作，加快4个外贸转型升级基地建设。落实进口货物贴息政策，进一步扩大进口规模。推动区域合作，积极对接京津冀、长江经济带、粤港澳大湾区等国家重大战略。深度融入沈阳经济区，强化产业融通链接、服务协同共享、基础设施互通、人才流动互用，大力培育中欧产业园，力争引进一批华晨宝马第三工厂配套项目，推进沈辽大道建设，打造与沈阳互动的高品质“生活圈”。加强与江苏南通对口合作，在新材料、智能装备、农业旅游等多个领域取得新进展。

　　（三）加速动能转换，激发新时代创新驱动内生动力

　　瞄准高质量发展方向，调整完善全市产业发展规划，构建“3+3+X”发展格局，着力打造芳烃及精细化工、铝材深加工、装备制造及汽车零部件“三大主导产业”，突出发展冰雪温泉、皮装裘皮、袜艺鞋业“三个特色产业”，积极培育文化旅游、健康养老、数字经济、“互联网+”、人工智能应用等新业态、新经济，促进产业集中、集聚、集约发展，为“智慧辽阳”建设注入新动能。

　　深入实施创新驱动战略。深化“政产学研用”协同创新，与东北大学等6家高等院校和科研院所签订全方位战略合作协议，促成科技成果转化40项。加快华信钢铁共性技术创新中心、奥克集团国家重点实验室辽阳分中心和精细化工成果转化中试基地建设。培育3个省级众创空间和星创天地。依托沈阳高水平“双创”孵化平台建立研发中心，吸引人才和技术为我所用。推动企业自主创新，开展高新技术企业提质扩量行动，新增高新技术企业15家以上，提高研发经费占地区生产总值比重。实施高层次人才特殊支持计划，培育一批“本土”科技型领军人才，打造一批“辽阳工匠”。推动高职院校与产业发展有效衔接，开展“冠名培养”和“订单培养”。

　　加快优化工业结构。全面推进制造业高质量发展。深度开发“原字号”。实施高性能聚丙烯等20个重点项目，做精做优芳烃及精细化工产业基地。加快铝板带箔等项目建设，促进铝材深加工系列项目产能逐步释放，做大做强铝合金精深加工产业基地。全力支持新型盾构机、航空密封件、高压共轨等项目建设，加速装备制造及汽车零部件产业发展。改造提升“老字号”。坚持“保大控小、保优汰劣、上新下旧”，推进特高压输变电出线装置、制药机械生产线等升级改造项目建设，加快钢铁产业转型步伐，进一步化解水泥、建材等行业过剩产能，坚决防止“地条钢”死灰复燃。培育壮大“新字号”。加速“两化”融合，启动建设欧洲智能制造小镇项目，积极培育工业互联网示范企业。深入实施消费品工业“三品”工程。

　　提升现代服务业发展水平。鼓励大型工业企业发展生产性服务业，加快忠旺国家重点实验室和总部大厦建设，支持辽化筹建精细化工中试基地。培育壮大金融业，新引进一批金融机构。加强与京东等大型电商平台合作，发展线上，巩固线下，进一步拓展企业销售渠道。推进辽阳现代综合物流园区建设，加快实施专业市场搬迁改造工程。完善城乡配送网络，推进农产品储藏库和物流服务平台建设，设立乡镇供销综合服务中心8个、村级综合服务社200个以上。培育养老产业，加快全国养老服务业综合改革、医养结合、居家和社区养老服务改革3个试点市建设。扩大住房消费，办好房交会，促进房地产销售。发展楼宇经济、夜经济和会展经济，打造历史文化、美食文化等4个特色街区。

　　大力发展旅游业。突出文化特色、产业特色、地域特色，打造具有丰富文化内涵的旅游项目。充分发挥旅游集团和双河建设集团作用，促进文化和旅游产业融合发展。开展皮装裘皮个性化定制，启动时尚产业园建设，加快向高端化、品牌化迈进。创新营销方式，建成电商直播中心，培育佟二堡皮装跨境电商平台，激活佟二堡千亿元市场发展新动能。完善弓长岭温泉集聚区配套设施，加快奥特莱斯温泉度假小镇等项目建设，打造国家级温泉度假区。坚持“冰天雪地也是金山银山”的理念，促进冬季旅游提质升级。进一步拓展旅游路线，开发市域、省域系列旅游产品。鼓励和规范民宿经济发展。

　　（四）发展现代农业，大力促进新时代乡村全面振兴

　　坚持农业农村优先发展，实施乡村振兴五年战略规划，推进农业供给侧结构性改革，促进农业增效、农民增收、农村增绿。

　　加快国家现代农业示范区建设。巩固提升10个现代农业示范区（带）建设成果，开展“多园一体”创建工作，实施138个现代农业精品项目。大力发展高效农业、绿色农业、休闲农业、认养农业，促进农村一二三产业融合发展。基本完成农村集体产权制度改革，全面完成农村土地承包经营权确权登记颁证工作。加快土地流转，大力培育新型农业经营主体，组建千亩以上土地股份合作社20家。划定玉米、水稻生产功能区和大豆生产保护区175万亩，调减玉米种植面积5万亩，力争完成万亩设施农业建设任务。粮食产量稳定在100万吨左右。深化农家科普大院和科技小院建设，耕种收综合机械化率达到86.5%。调整养殖业模式，科学规划生猪养殖布局，发展草食性畜牧业。加快发展农产品加工业，再实施一批农业产业化项目，推动佟二堡争创省级农产品加工集聚区。开展农业品牌提升行动，培育一批特色农产品品牌。有效防控非洲猪瘟、松材线虫等重大动植物疫病。

　　完善农业农村基础设施。新建高标准农田10万亩。深入实施太子河上游左岸生态综合治理工程。完成大中型灌区改造等水利工程建设年度任务。全面落实“河长制”“警长制”，集中开展河湖“清四乱”专项行动。推进“四好农村路”建设，大中修、改扩建各级公路500公里。实施农村饮水安全提升工程，惠及群众8万人。深入开展农村人居环境整治三年行动，有效治理农村生活垃圾、厕所粪污、生活污水，加强村庄规划管理，完善建设和管护机制，进一步改善村容村貌。

　　培育壮大县域经济。深入落实县域经济发展三年行动计划，推动辽阳县、灯塔市综合实力晋位升级。探索建立涉农资金统筹整合长效机制，积极开展试点工作，提高资金使用效率。完善县乡财政管理体制，全面推动“飞地经济”，激发乡镇发展活力。扶持壮大薄弱村集体经济。加快特色小镇建设，进一步完善基础设施，做大做强主导产业，深度挖掘历史文化、民俗风情、自然景观等旅游资源，提升小镇知名度。

　　（五）加强生态建设，巩固提升新时代绿色发展优势

　　坚持“绿水青山就是金山银山”的理念，全面落实“大气十条”“水十条”“土十条”，深入打好污染防治攻坚战，持续巩固“美丽辽阳”建设成果。

　　争创国家森林城市。荒山造林3万亩，绿化村屯105个，发展林下经济2000亩。综合整治非煤矿山地质环境，实施12个闭坑矿山生态治理项目，有序推进双河地区17个地块土地修复工程。

　　加强水污染治理。大力整治南地河等9条河流，力争7个国控河流断面全部达标。推进污水处理厂扩容提标，加快改造城乡污水管网，提高污水收集和处理率。强力治理畜禽养殖业污染，全面加强集中式饮用水水源地保护和规范化建设。

　　打赢蓝天保卫战。加快淘汰建成区20吨及以下燃煤锅炉，基本取缔禁燃区散煤销售网点。执行重点控制区大气污染物特别排放限值，全面管控城市扬尘和机动车尾气排放。实施最严格的秸秆禁烧制度，秸秆综合利用率达到87%。

　　扎实开展土壤防治。推进土壤环境质量监测工作，采集重点行业企业用地污染状况基础信息，初步确定土壤调查布点采样清单。

　　划定生态保护红线，统一确权登记红线内自然资源，确保完成“三线一单”技术报告编制工作，推动建立生态保护补偿机制。做好第三次全国国土调查工作。深入开展领导干部自然资源资产离任审计。全面完成中央环保督察“回头看”整改任务。

　　（六）坚持建管并重，聚力塑造新时代城市形象品质

　　创建全国文明城市和国家历史文化名城，建设有特色、有档次、有品位、有温度的“四有”城市，全面提升“文明辽阳”建设水平。

　　坚持精确规划。以规划引领城市建设，实现城市科学、良性、可持续发展。加快推进“多规合一”，有机整合历史文化名城规划、城市控详规划、重点区域和节点城市设计。明晰城市主色调，统筹区域建筑风格，彰显古城历史文化底蕴和城市风貌。

　　突出精致建设。依托历史文化街区和历史风貌区，规划建设独具特色的复古街区。完善交通体系，新建改造主次干道和街巷道路15条，延伸东兴路至宏伟区，确保辽庄线竣工通车，推进大型人防公交枢纽和河东交通综合枢纽二期工程，启动辽阳火车站西站房建设。提升生态品质，高标准实施绿化美化亮化工程，改造科普公园，治理青年湖公园水体，整治城市裸露地面，推进城市雨污分流和绿色除雪工作，实施垃圾焚烧发电项目。加强服务配套，完善燃气、供水、供电、供暖、环卫、停车等基础设施。

　　强化精细管理。破解城管执法体制机制瓶颈，建立多部门联动机制，提升城管执法效力。实施新运大街公交优先示范工程，提高城市公交出行分担率。进一步完善数字化城市管理系统，扩大网格化管理覆盖范围。启动环卫市场化改革。综合整治再生资源回收乱象、违章建筑、交通拥堵等问题，提高城市智慧化、精细化、社会化管理水平。

　　（七）着力改善民生，共享新时代辽阳振兴发展成果

　　坚持以人民为中心的发展思想，打造为民、便民、利民、惠民的公共服务体系，办好22项重点民生实事，推动“幸福辽阳”建设实现新突破。

　　坚决打好脱贫攻坚战。加快推进产业扶贫、精准医疗扶贫等“五个一批”重点工程，确保建档立卡贫困人口全部脱贫。开展脱贫质量“回头看”，确保“返贫”动态为零。

　　提供更可靠的社会保障。新增城镇就业1万人，力争贫困家庭大学生100%稳定就业。继续推进社保扩面征缴，新增五险扩面2.6万人。城乡低保、特困供养标准均提高6%以上。改造老旧小区12个、农村危房320户，维修老旧楼房60栋，实施“暖房子”工程5万平方米。实现低收入居民住房保障应保尽保。确保养老金按时足额发放。

　　办好人民满意教育。深入推进协作化办学，打造“三通两平台”升级版，推动优质教育资源开放共享。促进义务教育高质量均衡发展，确保辽阳县、灯塔市通过国家义务教育均衡验收。实施“学前教育追赶计划”，建成8所乡镇公办幼儿园，实现公办幼儿园乡镇全覆盖。完成“薄弱学校改造五年计划”，加快改善乡村小规模学校和乡镇寄宿制学校基础设施。实施“名师、名校、名校长”领航工程，加强师德师风建设。整治校园周边环境，创建“平安校园”。

　　丰富群众文化体育生活。深入实施文化惠民工程，推进基层公共文化服务体系建设，加强文化遗产保护和利用。深入挖掘乡土文化资源，实施“文化名人”培育工程，打好文化体育牌，开展丰富多彩的文体活动。做大做强文体产业，市场化运作各类文化演出和体育赛事。

　　提高医疗卫生服务水平。强化公共卫生体系建设，提升传染病防控能力。实施“名医、名科、名院”工程。以市中心医院为依托，建设互联网医院。加快推进乡镇卫生院标准化建设。

　　加强社会综合治理。优化升级社会治安防控体系，深入开展扫黑除恶专项斗争，切实提升平安建设能力。夯实信访工作责任制，信访存量和矛盾增量均减少30%以上。落实安全生产监管责任和企业主体责任，整治重点领域安全隐患，提高事故防控和应急救援能力。加强全民国防教育和国防动员建设。加快推进城乡社区治理创新。

　　（八）强化自身建设，全力打造新时代人民满意政府

　　加强政治建设。树牢“四个意识”，坚定“四个自信”，坚决做到“两个维护”。用习近平新时代中国特色社会主义思想武装头脑、指导实践、推动工作，确保党中央、国务院和省委、省政府部署要求在辽阳落地生根、开花结果。

　　加强民主法治建设。主动接受市人大及其常委会法律监督、工作监督，提高人大代表议案、建议办理质量。自觉接受市政协民主监督，积极支持政协参政议政，认真办理政协委员提案。充分听取各民主党派、工商联、无党派人士和工会、共青团、妇联等人民团体意见建议。坚持依法行政，重视和配合地方相关立法工作，推进法治政府建设。

　　加强作风建设。有效落实“重强抓”专项行动，深化“五包五促”，开展“千名干部包千企”活动。充分发挥12345市长热线与8890政务便民服务平台作用，着力解决好群众关心的物业管理、停车泊位等热点难点问题。弘扬“钉钉子、马上办”精神，勇于担当，善于攻坚。扎实做好第四次全国经济普查，提高政府科学决策水平。大兴调研之风，决策在一线制定，工作在一线落实，问题在一线解决。细化完善绩效考核管理办法，分出档次、拉开差距，激发广大干部工作热情。坚持说实话、出实招、求实效，打造务实高效政府。

　　加强廉政建设。落实全面从严治党主体责任，持之以恒正风肃纪。严格落实中央八项规定精神和省、市委实施细则，巩固拓展整治“四风”成果。加强审计监督，规范财政资金使用。深入推进政府系统党风廉政建设和反腐败斗争，努力营造政治清明、政府清廉、干部清正、政商关系亲清、同事关系清新的良好风气。

　　各位代表！新时代是奋斗者的时代，让我们高举习近平新时代中国特色社会主义思想伟大旗帜，在省委、省政府和市委的正确领导下，奋发图强，奋勇争先，奋力开创各项工作新局面，为全面建设“四个辽阳”，实现新时代辽阳全面振兴发展作出新的更大的贡献，以优异成绩向新中国成立70周年献礼！
